# The influence of a blended, theoretically-informed pre-implementation strategy on school-based clinician implementation of an evidence-based trauma intervention

**DOI:** 10.1186/s13012-019-0905-3

**Published:** 2019-05-30

**Authors:** Aaron R. Lyon, Clayton R. Cook, Mylien T. Duong, Semret Nicodimos, Michael D. Pullmann, Stephanie K. Brewer, Larissa M. Gaias, Shanon Cox

**Affiliations:** 1 University of Washington, 6200 NE 74th Street, Suite 100, Seattle, WA 98115 USA; 2 University of Minnesota, 250 Education Sciences Bldg, 56 East River Road, Minneapolis, MN 55455 USA; 3 Committee for Children, 2815 2nd Ave #400, Seattle, WA 98121 USA

**Keywords:** Individual determinants, Implementation strategy, Theory of planned behavior, Trauma intervention, Behavioral intentions, Adoption

## Abstract

**Background:**

Individual-level implementation determinants, such as clinician attitudes, commonly influence the successful adoption of evidence-based practices, but few explicit strategies have been tested with regard to their ability to impact these key mechanisms of change. This paper reports on an initial test of a blended, theoretically informed pre-implementation strategy designed to target malleable individual-level determinants of behavior change. Beliefs and Attitudes for Successful Implementation in Schools (BASIS) is a brief and pragmatic pre-implementation strategy that uses strategic education, social influence techniques, and group-based motivational interviewing to target implementation attitudes, perceived social norms, perceived behavioral control, and behavioral intentions to implement among mental health clinicians working in the education sector.

**Methods:**

As part of a pilot trial, 25 school mental health clinicians were randomized to BASIS (*n* = 12) or an attention control placebo (*n* = 13), with both conditions receiving training and consultation in an evidence-based intervention for youth experiencing trauma (the Cognitive Behavioral Intervention for Trauma in Schools). Theorized mechanisms of change (attitudes, perceived social norms, perceived behavioral control, and behavioral intentions) were assessed at baseline, post-training, and 4-month follow-up. Clinician participation in post-training consultation and intervention adoption were also tracked.

**Results:**

A series of regression models and independent sample *t* tests indicated that BASIS had significant, medium to large effects on the majority of its proximal mechanisms from baseline to post-training. BASIS was also associated with a greater latency between initial training in the intervention and discontinuation of participation in post-training consultation, with clinicians in the BASIS condition persisting in consultation for an average of 134 days versus 32 days for controls, but this difference was not statistically significant. At 4-month follow-up, most differences in the theorized mechanisms had attenuated, and approximately the same small number of BASIS clinicians adopted the trauma intervention as controls.

**Conclusion:**

Findings suggest that the brief BASIS pre-implementation strategy had a significant influence on its proximal mechanisms of change, but that these changes did not persist over time or translate into adoption of the trauma intervention. Implications for theory refinement, revisions to the BASIS protocol, and next steps for research surrounding individual-level implementation strategies are discussed.

**Trial registration:**

ClinicalTrials.gov Identifier: NCT03791281. Registered 31 December 2018—Retrospectively registered

**Electronic supplementary material:**

The online version of this article (10.1186/s13012-019-0905-3) contains supplementary material, which is available to authorized users.

## Background

To address persistent gaps in the use of evidence-based practices (EBPs), growing research has focused on the identification of specific implementation strategies, defined as methods or techniques used to enhance the adoption, implementation, and sustainment of a clinical program or practice [1, 2]. The current paper describes findings from an initial test of a theory-driven implementation strategy designed to target malleable individual-level determinants of behavior change (e.g., intentions to implement) among mental health clinicians in the education sector.

### Implementation strategies, determinants, and mechanisms

Implementation strategies may be discrete, involving one specific process or action; multifaceted, including a combination of two or more discrete strategies; or blended, which are multifaceted strategies that have been explicitly protocolized or branded [3]. To be optimally effective, strategies should be selected and applied based on the specific multilevel determinants (i.e., barriers and facilitators [4]) they are intended to target [1, 5]. Although the importance of inner setting (i.e., organizational) determinants is well established [6, 7], there are individual-level barriers (e.g., beliefs and attitudes) that commonly impede implementation outcomes [8–10]. Some studies have found that individual factors (especially attitudes) may be significantly more predictive of EBP use than organizational factors [11]. Further, while some organizational strategies have yielded encouraging results [12, 13], they are often time consuming and expensive [14]. Because implementation rests on the motivation, decisions, and behavior change of individuals within systems [15], it is critical to develop pragmatic (i.e., low-resource and contextually-appropriate) individual-level implementation strategies to target these determinants of implementation outcomes.

An important step of implementation strategy development is articulating their theorized mechanisms of change [5], although these are rarely evaluated [16, 17]. Theory is currently the best pathway through which to identify implementation mechanisms [5]. At the individual level, the theory of planned behavior (TPB) [18–20] has increasingly been applied to implementation behaviors [21, 22]. The central tenet of TPB is that one of the best predictors of behavior is a person’s behavioral intentions [18, 20], defined as motivations or conscious plans to exhibit particular behaviors. Behavioral intentions, in turn, are a function of an individual’s attitudes (cognitive appraisals of the behavior in question), subjective norms (an individual’s own estimate of the social pressure to perform the behavior), and perceived behavioral control (the extent to which an individual feels confident about being able to perform the behavior).

A recent meta-analysis of the TPB yielded an average effect size of .50 across a variety of patient behaviors (e.g., adherence to medical regimens) [23, 24]. The TPB is also the most commonly used social-cognitive theory for designing and evaluating the impact of implementation strategies [22]. The results of an implementation-oriented systematic review [25] suggest that the variance in clinician behavior explained by intentions is similar to that reported among patients, although very few studies have evaluated TPB constructs as mechanisms of behavior change.

### Beliefs and attitudes for successful implementation in schools

**Fig. 1:**
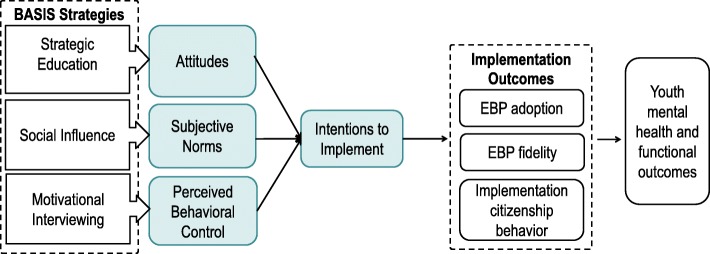
BASIS components aligned with TPB hypothesized mechanisms of change and implementation outcomes. Colored boxes reflect the theory of planned behavior components

Grounded in the TPB, Beliefs and Attitudes for Successful Implementation in Schools (BASIS) is a blended implementation strategy developed to target individual-level determinants of behavior change among school-based mental health clinicians. One in five youth experience a mental health problem [26] and 70–80% of youth mental health services are provided in schools [27–30]. Nevertheless, school mental health services are frequently not based on evidence for effectiveness [31–34]. Recent evidence shows that school mental health professionals’ intentions to use EBP are strongly associated with subsequent use [35] and that implementation may be driven as much or more by individual-level determinants (e.g., attitudes) than by organizational processes [11]. BASIS employs strategies targeting each TPB component: (1) strategic education about EBP and intervention fidelity to improve attitudes toward EBP, (2) social influence techniques to alter perceptions of subjective norms, and (3) motivational interviewing techniques to enhance perceived behavioral control. Figure 1 displays the core BASIS components, as well as their respective mechanisms of change (described in detail in “Methods” section). In practice, BASIS is a relatively brief (3–4 h) interactive session delivered to providers as an adjunct to training and consultation. Although most strategies tend to focus on the active implementation phase [36], BASIS is conceptualized as a pre-implementation strategy, delivered during the preparation phase, prior to and immediately after training and before consultation [6]. BASIS is not intended to replace other organizational implementation supports, such as coaching, high-quality training, and leadership. Rather it is designed to be compatible with and facilitative of other organizational (e.g., improving leadership) and innovation-specific (e.g., ongoing professional development connected to professional learning communities) implementation supports.

#### Evaluations of previous versions of BASIS

When delivered alongside training in a universal mental health EBP, a preliminary version of BASIS was associated with significantly more favorable attitudes toward EBP among 1181 teachers and administrators in a pre-post trial (*d* = 1.03; [37]). Attitudes, in turn, were associated with two measures of intervention integrity (*d* = .51 and *d* = .67). Nevertheless, the preliminary version was relatively long and designed to target educator (rather than clinician) delivery of EBP. To address these limitations, our research team recently adapted BASIS for school mental health clinicians and found that it was perceived to be feasible, appropriate, and likely to have an impact on proximal mechanisms of behavior change [38].

### Study aims

The current paper reports on a National Institute of Mental Health-funded study designed to conduct an initial randomized trial of the revised BASIS implementation strategy with a sample of school mental health clinicians, to augment training and consultation in a leading evidence-based trauma intervention (Cognitive Behavioral Intervention for Trauma in Schools; CBITS) [39]. Although trauma is a growing concern among many educators and parents [40, 41], evidence-based interventions for youth exposed to trauma are rarely available [41]. High levels of clinician drop-out from, or discontinuation of, CBITS implementation efforts are a significant problem [42]. Evidence suggests that only 31% of sites follow through with delivery of group sessions after receiving training and consultative support. In the current study, we hypothesized that clinicians randomized to BASIS would demonstrate greater changes in target TPB mechanisms (i.e., attitudes, social norms, perceived behavioral control) from pre-training to post-training (hypothesis 1), would demonstrate higher intentions to implement (hypothesis 2), and would demonstrate higher rates of CBITS adoption and sustained participation in consultative implementation support activities (hypothesis 3). We also had an exploratory research question surrounding the extent to which changes in mechanisms would sustain from post-training to end of year follow-up (research question 1). Finally, because our scientific questions related to individual-level determinants and processes, we did not include organizational implementation supports beyond training and consultation. This was intentional to isolate the influence of BASIS on hypothesized individual-level mechanisms of action and facilitate a nuanced understanding of how they function to support implementation. However, we evaluated aspects of implementation climate—an organizational-level variable—as an additional check on the comparability of the BASIS and control groups to rule out alternative explanations for any group differences.

## Methods

To address the study aims, we conducted a pilot randomized trial (randomization at the clinician level) of the impact BASIS, relative to an attention control, on CBITS adoption.

### Participants

**Fig. 2:**
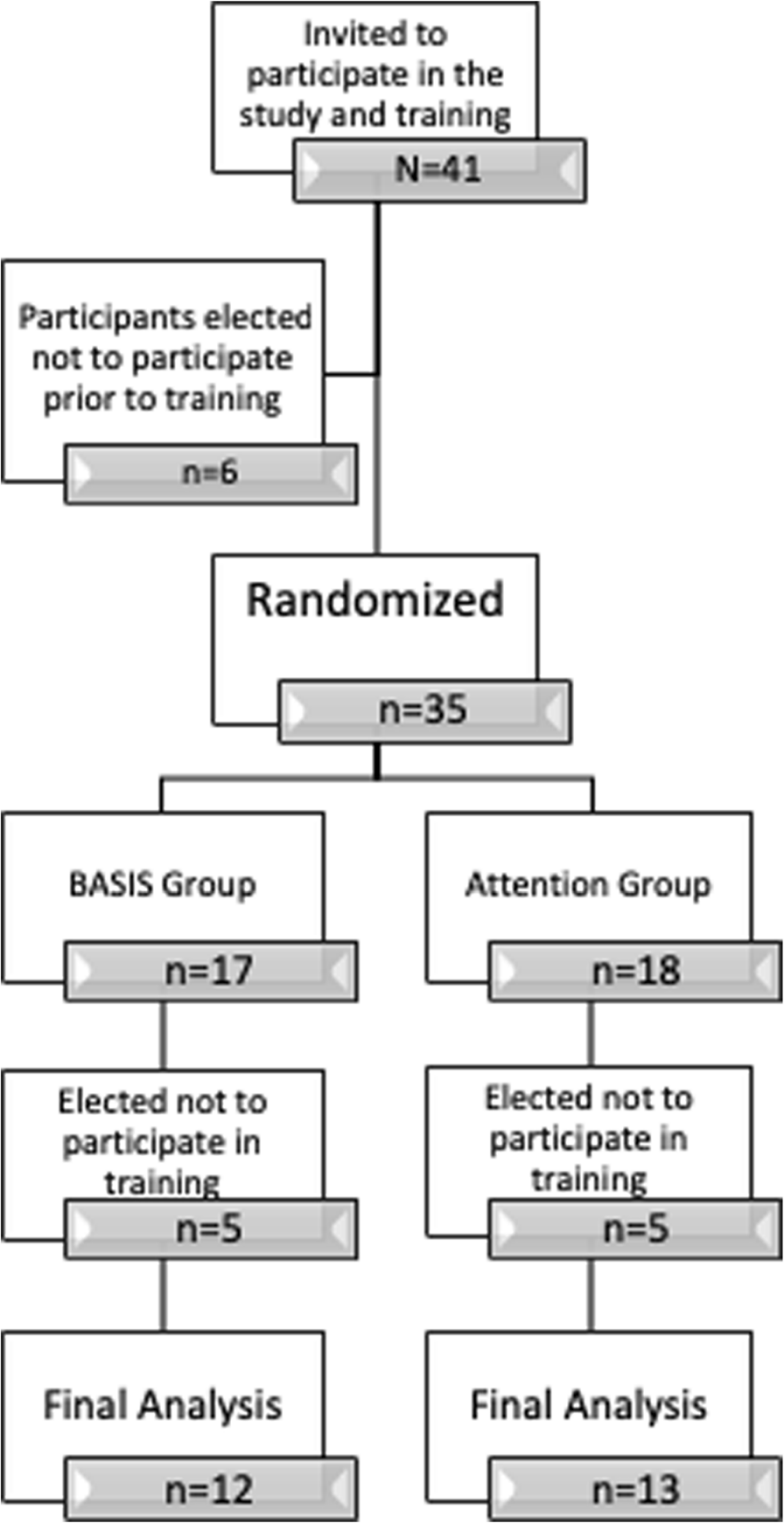
CONSORT diagram for study participation

Mental health clinicians from two school districts in the Pacific Northwest participated. These two districts were selected based on their interest in integrating trauma-informed services into secondary schools. Out of the 41 providers who were invited, 35 agreed to participate and were randomized. Following randomization, ten participants elected not to participate, leaving a final pool of 25 school mental health providers (12 in the BASIS condition, 13 in the control) attending the trainings (see CONSORT diagram; Fig. 2). This sample size was determined to be adequate for a preliminary trial, based on effect sizes documented in previous work [37] and the constraints of the funding mechanism. Table 1 displays participant demographics for the overall sample and stratified by condition, with χ^2^ analyses to test for condition differences.

**Table 1:** Demographic comparisons of the two groups

	Total		BASIS group *N* = 12		Control group *N* = 13		χ^2^	*p*
	*N*	%	*N*	%	*N*	%		
Gender								
Female	20	80.0%	10	83.3%	10	76.9%	0.031	0.859
Male	5	20.0%	2	16.7%	3	23.1%		
Race								
Asian	1	4.0%	1	8.3%	0	0.0%	2.72	0.605
Black	5	20.0%	2	16.7%	3	23.1%		
Hispanic	1	4.0%	0	0.0%	1	7.7%		
Native	0	0.0%	0	0.0%	0	0.0%		
White	16	64.0%	8	66.7%	8	61.5%		
Multi-racial	2	8.0%	1	8.3%	1	7.7%		
Age								
18 to 24 years old	1	4.0%	0	0.0%	1	7.7%	2.62	0.624
25 to 34 years old	5	20.0%	2	16.7%	3	23.1%		
35 to 44 years old	7	28.0%	4	33.3%	3	23.1%		
45 to 54 years old	7	28.0%	2	16.7%	5	38.5%		
55 to 64 years old	3	12.0%	2	16.7%	1	7.7%		
Missing	2	8.0%	2	16.7%	0	0.0%		
Grade-level								
Middle school	13	52.0%	6	50.0%	7	53.8%	0.04	0.582
High school	12	48.0%	6	50.0%	6	46.2%		

### Procedures

Recruitment flyers were sent via email to all eligible school mental health staff (i.e., those with credentials to provide mental health services) from the participating districts. Interested clinicians were contacted via phone, consented, and emailed a link to the pre-training survey using the Qualtrics online platform. Based on the pre-training survey, participants were assigned to condition via a nearest neighbor analysis using variables for grade level, EBP attitudes, social norms, perceived behavioral control, and intentions to implement EBPs, (see “Measures” section below) to identify matched pairs. Pairs were identified via the best match as identified by nearest Euclidean metrics, with a fixed *k* of 2; in situations where three or more cases had competing best distances, the match was randomly selected.

Online pre- and post-training surveys were collected from both the attention control (AC) plus CBITS and the BASIS plus CBITS conditions. The CBITS training was then followed by biweekly consultation for 4 months with clinician participation tracked. To prevent condition contamination, training and consultation was provided separately (but using the same trainers) for the two conditions. After the consultation period, all providers completed online post-intervention measures.

### Study conditions

#### Attention control

Providers randomly assigned to AC received a 3-hour AC intervention prior to CBITS training which was designed to control for dose, information provided, and interventionist effects. The AC facilitator defined, described, and advocated for EBP implementation in schools. Content was didactic, as is typical in trainings for school mental health providers.

#### Basis

The BASIS condition consisted of pre- and post-training sessions that bookended the CBITS training (see below). In this condition, clinicians received the three-hour group-based, interactive BASIS strategy, delivered by the same research team member who delivered the AC training to control for facilitator effects.

### BASIS implementation strategy

Throughout BASIS, three components are embedded into the didactic sections and interactive activities, designed to target the three elements of TPB (see Table 2). These components are described below in accordance with guidelines for implementation strategy reporting [2].

**Table 2:** BASIS components and content

Component	Content
Strategic education (attitudes)	1. Connecting EBP to student success 2. Recognizing vulnerabilities to adopt non-EBPs 3. Address common myths about EBP 4. Evaluating evidence for practices 5. Promoting understanding of fidelity of EBP
Social influence (social norms)	1. Providing normative information 2. Testimonials from experts 3. Testimonials for similar others 4. Evoking public commitments
Motivational intervention (perceived behavioral control)	1. Professional values clarification 2. Pros and cons activity to elicit change talk 3. Anticipate implementation barriers 4. Values-directed implementation goals 5. “Ruler questions” (e.g., how confident?)

#### BASIS component 1: strategic education about EBP and intervention fidelity to improve attitudes toward EBP

BASIS incorporates strategic education to (1) help clinicians learn about the benefits of EBP for them professionally and for closing gap in access to high-quality services for the youth they serve, as well as (2) alter any previously held beliefs they may have had about negative outcomes associated with EBP [43]. For example, clinicians estimate the potential access gap in their home schools and learn how delivery of EBPs is integral to addressing that gap. In addition, definitions and dimensions (e.g., adherence and dosage) of fidelity are presented, and clinicians are prompted to reflect on the critical importance of fidelity across a range of professions (e.g., engineering, farming, aviation). Moreover, clinicians learn about the outcomes of popular but ineffective practices (e.g., bloodletting, fat free diets) and how to recognize cognitive “shortcuts” that enhance individual vulnerability to adopting non-EBPs.

#### BASIS component 2: social influence techniques to alter perceptions of subjective norms

Recognizing that education alone is unlikely to change professional behavior, BASIS also relies on evidence-based social influence techniques. In particular, two strategies are used: (1) social proofing messages (“social proofs”) that use data or testimonials to describe the behavior or attitudes of others, and (2) strategies to induce cognitive dissonance. Social proofs have been effectively used to reduce problem behaviors including alcohol use [44], illegal drug use [45], cigarette smoking [46], and eating disordered behaviors [47]. Evidence suggests that social proofs are most influential when people are given information about the current behavior of individuals with whom they closely identify. In BASIS, normative data and testimonials are used to validate clinician experiences of EBP implementation barriers (e.g., lack of time, low administrative support), and model commitments to problem-solving these barriers. In addition, expert testimonials address common myths about EBPs (e.g., that they are inflexible).

Strategies to induce cognitive dissonance operate on the premise that individuals strive for consistency between their attitudes and actions. Thus, desired behaviors can be increased by evoking commitments that are active (rather than passive), public (rather than private), and voluntary (rather than coerced) [48, 49]. In BASIS, clinicians set public goals for the upcoming training and for EBP implementation, and collaboratively generate potential solutions to overcome common barriers to implementation (e.g., time; lack of supervisor support). Clinicians’ ideas are compiled and they are told their ideas will be shared with other clinicians who may encounter similar barriers. This activity is intended to position clinicians to freely and publicly advocate for potential solutions to EBP implementation.

#### BASIS component 3: motivational interviewing techniques to enhance perceived behavioral control

Motivational interviewing (MI) is a nondirective, person-centered counseling style for helping patients to explore and resolve ambivalence [50]. Decades of research have demonstrated significant effects of MI on adult and youth health behaviors [51–54] and on EBP implementation among teachers and primary care providers [55, 56].

The BASIS facilitator utilizes group MI techniques by adopting an empathic, supportive, and nondirective style to elicit self-motivational statements, encourage the elaboration of change talk, and enhance perceived behavioral control. For example, clinicians engage in an evidence-based values affirmation activity, which has been shown to decrease defensiveness toward change and enhance motivation to engage in value-congruent behavior [57]. Clinicians are asked to reflect on and share with the group reasons why they chose their profession and why they continue despite challenges. Clinicians also anticipate barriers that may arise in implementation, engage in collaborative problem-solving to brainstorm ways to address those barriers, answer standard MI “ruler” questions (e.g., “On a scale of 1-10, how confident are you in your ability to implement an EBP?”) [50], and engage in group discussion about what is needed to increase their ratings. Additionally, providers are asked to recall other times when they have successfully made changes in their careers, highlighting their capability to take on and implement innovative practices. Throughout the session, the facilitator uses standard MI techniques: elaborate on change talk, express empathy, roll with resistance, and emphasize autonomy.

### CBITS training and consultation

CBITS is an evidence-based intervention consisting of ten group sessions, 1–3 individual sessions, two parent psychoeducational sessions, and one teacher educational session. Used with students in grades 5–12 who have witnessed or experienced traumatic life events, CBITS has been shown to reduce symptoms of trauma and depression and improve academic outcomes [58, 59]. Candidate students are eligible for CBITS based on screening procedures to identify trauma-exposed students from the entire population of students in a school who are in need of and would benefit from CBITS. CBITS was developed to be used by school mental health clinicians, has established training protocols, and provides structured post-training consultation supports [60, 61].

After receiving BASIS or AC, all providers participated in a standard, 1.5-day CBITS training delivered by the same certified trainers, blinded to condition. CBITS training included best practices for educational meetings: didactic content delivery, rehearsal activities, and performance-based feedback [62]. A trained CBITS consultant, also blind to condition, provided biweekly post-training group consultation to providers who received either BASIS and AC. Participation in post-training group consultation was an expectation of clinician enrollment in the study. Consultation groups (6–8 providers/each) were formed within BASIS or AC conditions to avoid contamination.

### Measures

A detailed description of all study measures, including reliabilities in the current sample, is provided in Additional file 1.

#### Attitudes

The school version of the Evidence-Based Practice Attitudes Scale (EBPAS) [63] is a 26-item adaptation of the original EBPAS [64]. Subscales include Requirements, Appeal, Openness, Divergence, Fit, and Burden. Attitudes were measured at baseline, post, and follow-up time points.

#### Perceived social norms

The modified subjective norms measure, based on guidelines for developing reliable and valid measures of theory of planned behavior (TPB) constructs [65, 66], captures two types of EBP implementation-related subjective norms: injunctive (what a social group would approve of) and descriptive (how a social group actually behaves). Perceived social norms were measured at baseline, post, and follow-up time points.

#### Perceived behavioral control

A modified version of the Teacher Self-Efficacy Scale [67] with ten items was used to assess perceived behavioral control in implementing EBP. Perceived behavioral control was measured at baseline, post, and follow-up time points.

#### Implementation citizenship

The School Implementation Citizenship Behavior Scale (S-ICBS) [68], modified from the original Implementation Citizenship Behavior Scale [69], measures clinicians’ perceptions regarding how school staff engage with EBPs within their specific school context. Subscales include Helping others, Keeping informed, Taking Initiative, and Advocacy. Implementation citizenship was measured at baseline, post, and follow-up time points.

#### Behavioral intentions to implement

The Modified Intentions to Use Scale [70] assesses school mental health providers’ intentions to implement EBP. This scale was developed based on established guidelines for developing behavioral intention measures using the TPB [65, 66] and was administered pre and post BASIS and AC conditions to examine changes in school mental health providers’ intentions to implement CBITS.

#### BASIS fidelity

An observational fidelity tool was developed by the research team to capture adherence to 33 total components of BASIS. Two coders independently rated a video recording of the BASIS condition; one rater coded 97% and the second rater coded 94% of 33 BASIS components as having been delivered. The raters failed to jointly classify any component as not delivered. In situations such as this, Cohen’s kappa statistic is an inappropriate measure of interrater reliability [72]. Therefore, we report that raters were in agreement on 91% of the components delivered in the BASIS condition, and that a minimum of 91% of components were delivered during BASIS. Only one rater coded the AC condition; it covered 21% of BASIS components.

#### Consultation engagement

Each participant was provided the opportunity to attend up to 13 consultation phone calls with a CBITS consultant. At the end of each call, the consultant recorded who attended the consultation call, whether or not participants completed homework (when applicable), and overall engagement in the call. Ongoing participation in consultation included (1) attending at least one post-training consultation session, (2) number (%) of consultation sessions attended, and (3) days post-training to consultation dropout.

#### Adoption

Adoption was operationalized as the initiation of a CBITS group at any point during study participation, the number of days until the first CBITS session, and the number of days until implementation dropout. Previous research indicates that the vast majority of CBITS groups, once initiated, are completed [42].

#### Implementation climate

The school-adapted Implementation Climate Scale [68], modified from the original Implementation Climate Scale [71], measures clinician perceptions of the extent to which EBP implementation is expected, supported, and rewarded in their setting. Subscales include Focus on EBP, Educational Support for EBP, Recognition for EBP, Rewards for EBP, Use of Data to support EBP, Use of Existing Supports/Infrastructure for EBP Implementation, and Degree of EBP Integration. Because it was not theorized to change as a result of BASIS, implementation climate was measured at post-BASIS and follow-up time points. While implementation climate as a construct exists at the organization level, the construct reflects individuals’ reactions to the context of a particular organization and, therefore, also may be measured at the individual level.

### Data analytic approach

Means and standard deviations were calculated for all scales and subscales at baseline, post-training, and follow-up. For all variables collected at baseline and post-training, we computed a series of ordinary least square (OLS) regression models predicting post-training score, using baseline score as covariate and condition as a predictor. Similarly, for all variables collected at post-training and follow-up, we computed a series of OLS regression models predicting follow-up score, using post-training score as a covariate and condition as a predictor. Some variables were not collected at baseline; for these, we computed independent sample *t* tests to compare conditions at post-training. There was very little missing data: one person in the BASIS group skipped a section of the baseline survey, and therefore was missing data on the EBPAS, social norms, self-efficacy, and EBP intentions. No other participants were missing data. We imputed missing values for these variables using OLS regression models using all available data as predictors. We chose OLS regression rather than multiple imputation or maximum likelihood methods due to the small sample size, small amount of missing data, and relative ease of these calculations. Due to small sample size and limited statistical power, we present standardized coefficients (*β*s) and 95% confidence intervals (significant effects at *p* < .05 are highlighted to be consistent with convention), and we do not adjust for familywise error rate. Because these are standardized coefficients, they can be interpreted as effect sizes and compared across variables for strength of effect, which we interpret using .1 as a small effect (a tenth of a standard deviation), .3 as a medium effect (a third of a standard deviation), and .5 as a large effect (half of a standard deviation). Tables provide all results; in the section below, we report on all effects of *β* = .3 or larger.

## Results

### Hypothesis 1: pre- to post-training change in mechanisms

Table 3 depicts the unadjusted mean scores and standard deviations for all outcome measures at baseline, post-training, and follow-up, as well as standardized coefficients for intervention effects obtained via multiple regressions using baseline scores as covariates.

**Table 3:** Mean scores, standard deviations, and BASIS impact for outcomes at each timepoint

Measures	Condition	*n*	Baseline		Post		Follow-up	Baseline to post regression			Post to FU Regression	
			Mean	SD	Mean	SD	Mean	SD	Beta	95% CI	Beta	95% CI
Attitudes toward EBPs (EBPAS)												
Requirement	BASIS	11	8.85	2.40	9.45	2.81	9.89	1.17	.106	*−* .*211*, .*423*	.396	*−* .*010*, .*802*
	Attention	13	9.69	1.65	9.54	2.26	7.31	4.39				
Appeal	BASIS	11	12.74	3.35	14.18	1.78	13.33	2.18	.**489**	.*158*, .*820*	− .026	*−* .*379*, .*327*
	Attention	13	13.54	1.94	12.08	2.72	12.00	3.06				
Openness	BASIS	11	11.76	2.20	13.73	1.74	12.22	1.72	.**488**	.*174*, .*802*	− .121	*−* .*405*, .*163*
	Attention	13	13.00	2.48	12.00	2.83	11.62	3.15				
Divergence	BASIS	11	12.53	1.85	12.27	2.37	13.00	2.18	− .105	*−* .*517*, .*307*	.**481**	.*261*, .*701*
	Attention	13	13.31	1.44	13.00	1.83	12.08	1.38				
Fit	BASIS	11	23.03	3.76	24.18	2.93	22.56	3.88	.**473**	.*180*, .*766*	.191	*−* .*271*, .*653*
	Attention	13	22.31	3.84	19.77	4.51	19.31	3.99				
Burden	BASIS	11	10.70	2.45	10.18	3.46	9.11	3.95	.277	*−* .*143*, .*697*	.074	*−* .*361*, .*509*
	Attention	13	11.08	2.25	8.62	3.97	7.92	3.48				
Social norms												
Injunctive norms	BASIS	11	0.90	1.14	1.36	1.04	0.81	1.48	.309	.*721*, *−* .*103*	.357	.*102*, *−* .*817*
	Attention	13	1.00	0.82	0.81	0.75	1.42	0.62				
Descriptive norms	BASIS	11	1.11	1.26	1.45	0.76	0.64	0.90	.**474**	.*859*, .*089*	.348	.*102*, *−* .*798*
	Attention	13	1.21	0.89	0.60	1.04	0.69	0.90				
Self-efficacy	BASIS	9	35.11	6.07	35.44	4.28	31.78	5.45	.360	*−* .*020*, .*740*	− .297	*−* .*667*, .*073*
	Attention	13	34.85	4.76	31.08	5.81	32.08	5.62				
Implementation citizenship behaviors (ICBS)												
Helping others	BASIS	11	2.73	0.65	2.55	1.11	2.15	1.26	.166	*−* .*185*, .*517*	− .082	*−* .*424*, .*260*
	Attention	13	1.95	0.97	1.85	0.95	1.82	1.21				
Keeping informed	BASIS	11	2.85	0.70	2.91	0.67	2.41	0.98	.102	*−* .*163*, .*366*	− .275	*−* .*569*, .*020*
	Attention	13	2.51	1.18	2.49	1.22	2.64	1.13				
Taking initiative	BASIS	11	3.11	0.65	2.86	0.81	2.44	1.16	.087	*−* .*254*, .*428*	**−** .**271**	*−* .*493*, *−*.*050*
	Attention	13	2.60	0.90	2.48	0.89	2.58	0.98				
Advocacy	BASIS	11	3.36	0.54	3.15	0.84	2.69	1.24	− .053	*−* .*434*, .*327*	− .218	*−* .*439*, .*003*
	Attention	13	2.75	0.82	2.77	0.79	2.78	0.97				
Mean total	BASIS	11	3.01	0.51	2.87	0.79	2.42	1.09	.066	*−* .*244*, .*376*	**−** .**252**	*−* .*489*, *−* .*016*
	Attention	13	2.45	0.87	2.40	0.85	2.46	0.98				
Intentions to implement CBITS	BASIS	9	31.00	5.68	30.89	4.91	24.78	7.17	.**339**	.*007*, .*671*	− .154	*−* .*597*, .*289*
	Attention	13	29.54	4.82	24.08	8.37	24.08	7.92				

Bolded betas represent significant effects. Regression models examine impact of condition, controlling for baseline score

All outcomes for pre- to post-training that had medium or greater effect sizes were in the predicted direction. The attention control group did not improve from pre- to post-training on any variables, as indicated by raw score. Self-efficacy had a medium effect size with the BASIS group remaining stable while the AC deteriorated (*β* = .36, CI = − .02 to .74). Three of the six EBP attitudes subscales had a medium effect size, with the BASIS group showing an improving trend and the AC group showing deterioration for the subscales of Appeal (*β* = .489, CI = .159 to .820), Openness (*β* = .49, CI = .17 to .80), and Fit (*β* = .47, CI = .18 to .77). Both of the Norms subscales had a medium effect size, with the BASIS group showing an improving trend and the AC group showing deterioration for Descriptive (*β* = .47, CI = .86 to .09) and Injunctive Norms (*β* = .31, CI = .72 to − .10). None of the Implementation Citizenship Behaviors subscales had a medium or greater effect size.

### Hypothesis 2: intentions to implement

Intentions to implement showed a medium effect size, with the BASIS group remaining stable while the AC group deteriorated (*β* = .34, CI = .01 to .67).

### Hypothesis 3: consultation participation, engagement, and CBITS adoption

Eight out of 12 (66.7%) BASIS clinicians participated in at least one post-training CBITS consultation session, as compared to six out of 13 (46.2%) for AC; this difference was not statistically significant (χ^2^ = 1.07, *p* = .30). We restricted the following three analyses to only include those 14 individuals who attended at least one consultation session. The BASIS group attended a mean of 4.1 sessions and the AC attended a mean of 5.8, which was not statistically significant (*t*_(12)_ = .78, *p* = .45). The average engagement score for BASIS was 1.7 (*SD* = 16) and for AC was 2.5 (*SD* = 1.9), also not statistically significant (*t*_(12)_ = .91, *p* = .38). The average percentage of CBITS consultation homework assignments completed, after prorating for number of sessions attended, for BASIS was 27% (*SD* = 36%) and for AC was 43% (*SD* = 30%), also not statistically significant (*t*_(12)_ = .89, *p* = .39).

**Fig. 3:**
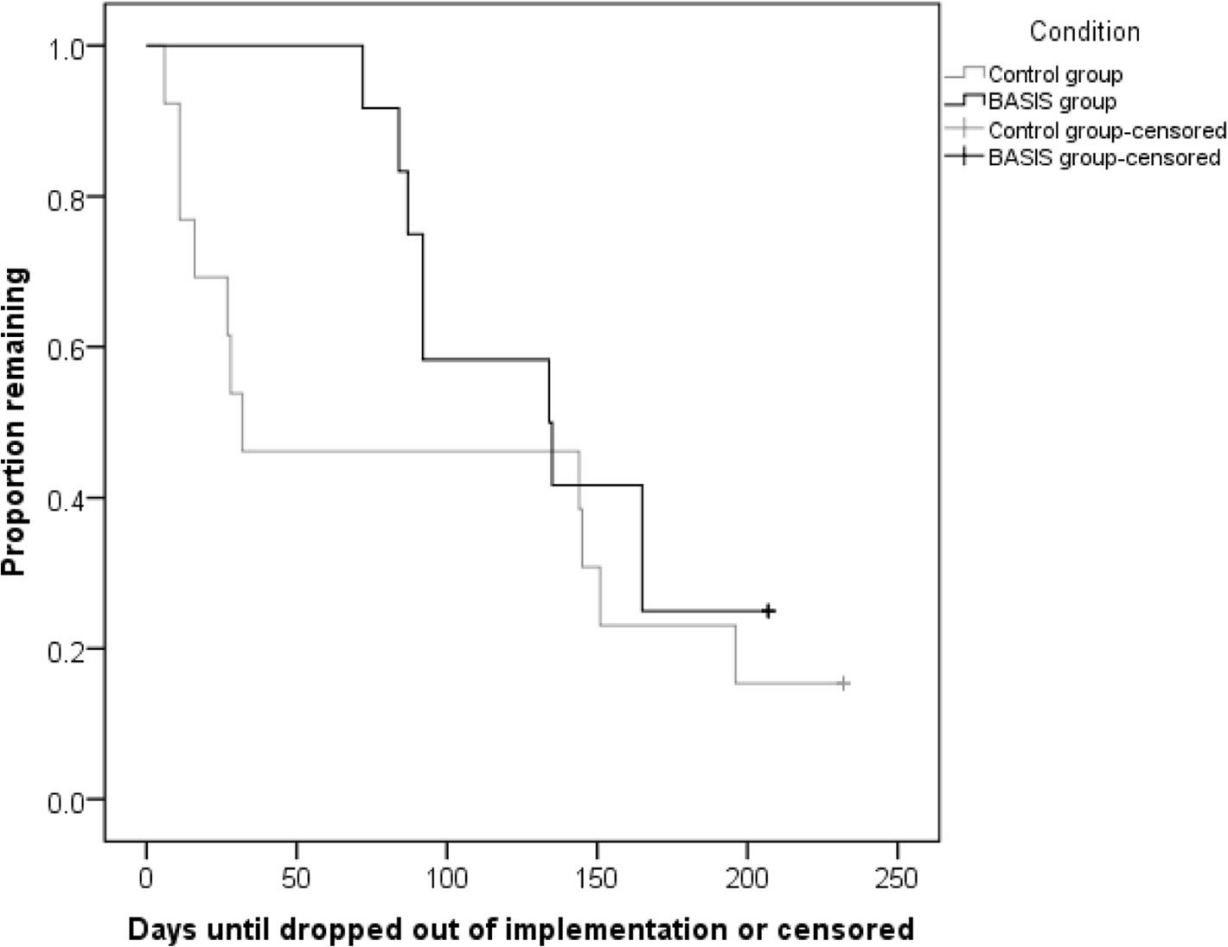
Time-to-event analysis: days until providers dropped out of CBITS implementation

**Fig. 4:**
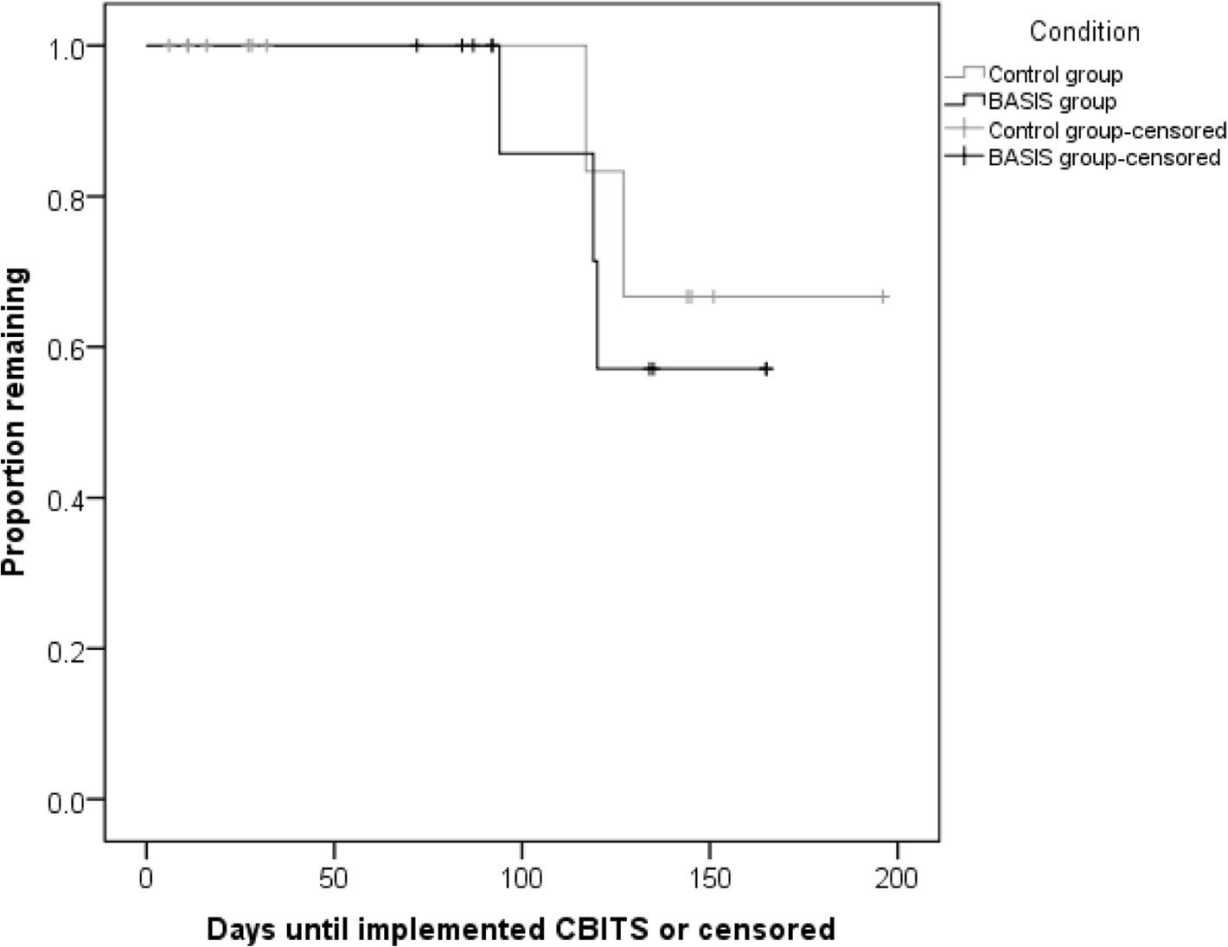
Time-to-event analysis: days until providers initiated a CBITS group

There were 15.4% (two of 13) attention control participants and 25.0% (3 of 12) BASIS participants who began a CBITS group during the study; this difference was not statistically significant (χ^2^ = .36, *p* = .55). A Kaplan-Meier time-to-event analysis (see Fig. 3) found that the median dropout from their commitment to implement a CBITS group was 32 days for the AC group, and 134 days for the BASIS group, which was large but not a statistically significant difference (log-rank χ^2^ = 1.08, *p* = .30; Breslow χ^2^ = 2.2, *p* = .14; Tarone-Ware χ^2^ = 1.6, *p* = .21). There were no significant differences in the number of days until a CBITS group began (log-rank χ^2^ = .18, *p* = .67; Breslow χ^2^ = .24, *p* = .63; Tarone-Ware χ^2^ = .21, *p* = .65), though medians could not be calculated because fewer than half of participants implemented CBITS (Fig. 4).

### Exploratory research question: post-training to end-of-year follow-up change in mechanisms

For post-training to the follow-up timepoints (Table 3), there were fewer effects of medium or greater size. Two of the EBP Attitudes subscales had a medium effect size. The strongest effects from post-training to follow-up were for EBPAS Divergence (*β* = .48, CI = .26 to .70). For this variable, higher scores indicate that participants are more likely to report that research-based interventions are clinically useful or as important as clinical experience. EBPAS Requirement also had a medium effect (*β* = .40, CI = − .01 to .80). For both EBPAS subscales, the BASIS group showed an improving trend while the AC group deteriorated. For Social Norms, both subscales had a medium effect size, including Injunctive (*β* = − .36, CI = .10 to − .82) and Descriptive Norms (*β* = − .35, CI = −  10 to − .80). For Injunctive Norms, the BASIS group deteriorated while the AC group showed stronger norms. For Descriptive Norms, the BASIS group deteriorated while the AC group remained stable. There were no other medium or greater effect sizes for any construct, including self-efficacy, implementation citizenship, or intentions to implement CBITS. However, one implementation citizenship subscale was statistically significant, Taking Initiative (β = − .27, CI = − .49 to − .05), and the total score for implementation citizenship was statistically significant (β = − .25, CI = − .49 to − .02). Finally, as can be seen in Table 4, implementation climate was comparable across BASIS and AC groups at post, with no statistically significant differences between the groups at either timepoint, and no effect sizes greater than small effects.

**Table 4:** Implementation climate means, standard deviations, difference across conditions, and BASIS impact from post to follow-up

Measure	Condition	*n*	Post		Follow-up		Post-training independent *T* test		Post to FU regression	
			Mean	SD	Mean	SD	*t*	*p*	Beta	95% CI
ICS focus	BASIS	11	2.67	0.87	2.33	1.02	− .905	.375	− .065	*−* .*522*, .*392*
	Attention	13	2.33	0.92	2.33	0.77				
ICS education	BASIS	11	2.52	0.89	2.23	0.85	− .961	.347	− .125	*−* .*596*, .*346*
	Attention	13	2.18	0.82	2.33	0.58				
ICS recognition	BASIS	11	2.73	0.61	2.10	0.74	− 1.136	.268	− .299	*−* .*754*, .*156*
	Attention	13	2.41	0.73	2.46	0.89				
ICS reward	BASIS	11	1.24	0.83	0.80	0.77	− .907	.374	− .139	*−* .*591*, .*313*
	Attention	13	0.97	0.62	0.90	0.77				
ICS data	BASIS	11	2.36	0.74	1.58	0.76	− 1.080	.292	− .160	*−* .*625*, .*305*
	Attention	13	1.94	1.10	1.73	1.02				
ICS support	BASIS	11	2.21	1.00	1.70	1.01	− .133	.896	− .067	*−* .*532*, .*398*
	Attention	13	2.15	1.13	1.82	1.07				
ICS integrate	BASIS	11	2.18	0.85	1.53	1.06	− 1.160	.258	− .141	*−* .*600*, .*318*
	Attention	13	1.77	0.88	1.62	0.80				
ICS total	BASIS	11	2.28	0.62	1.73	0.73	− 1.175	.252	− .149	*−* .*635*, .*337*
	Attention	13	1.96	0.71	1.87	0.61

## Discussion

The current study evaluated the influence of BASIS—a blended, individual-level pre-implementation strategy—on theorized mechanisms of behavior change, sustained participation in post-training implementation supports, and CBITS adoption. Findings suggested that, consistent with its underlying theory of change, BASIS had significant effects on the majority of its proximal outcomes, with BASIS clinicians demonstrating higher levels on target mechanisms relative to the attention control at post-training. This included medium to large effect sizes for attitudes (appeal, openness, fit), descriptive social norms, self-efficacy, and intentions to implement CBITS. Relative to the attention control, clinicians in the BASIS condition persisted in consultation over four times longer than controls (i.e., 134 versus 32 days), but this difference was not statistically significant. Evaluation of the mechanisms at the follow-up time point suggested that many changes in mechanisms did not persist over the full 4-month period. Further, BASIS did not demonstrate detectable effects on clinician adoption behaviors among the small number of clinicians who delivered the CBITS intervention (*n* = 5; three BASIS and two AC), indicating that post-training changes on the target individual-level determinants may be insufficient to influence implementation behaviors above and beyond training and consultation.

### BASIS and the theory of planned behavior

The impact of BASIS on the TPB mechanisms—particularly attitudes (appeal, openness, divergence, fit) and descriptive norms—and intentions to implement is aligned with theory and prior research. Similar to the current study, prior interventions that aimed to impact healthcare providers’ attitudes, subjective norms, perceived behavioral control, and intentions also found significant changes immediately post-training, but did not see sustained effects over time [73].

Unlike previous studies, however, we did not find evidence that these mechanisms facilitated delivery of CBITS. In a systematic review of the impact of social cognitive theories on healthcare professional’s intentions and behaviors, Godin and colleagues [22] found that 35% of the variance in behaviors across studies was associated with elements of the TPB. In particular, of all of the TPB mechanisms, healthcare professionals’ beliefs about their capabilities, including their perceived behavioral control (i.e., self-efficacy), was the most consistent predictor of behavior. In the current study, BASIS demonstrated a medium, but nonsignificant, effect on perceived behavioral control. It is possible that perceived behavioral control is an especially important component of the TPB that is essential to influence in order to facilitate behavior change and EBP implementation, but may be particularly challenging to impact in a time-limited pre-implementation intervention. Future implementation efforts may place more emphasis on enhancing this construct. In contrast, subjective norms are often considered the weakest or most inconsistent predictor of behavioral change [18, 74–76]. Although BASIS had moderate to strong impacts on both injunctive and descriptive norms at the post timepoint, this domain of the TPB may be necessary but insufficient to facilitate uptake and sustainment of an EBP. Furthermore, despite initial improvements, both types of social norms deteriorated for BASIS clinicians at the follow-up time point, reaching levels that were comparable to baseline or the control condition. As described below (see “Potential BASIS revisions” section), this suggests that BASIS, while time limited and efficient, might result in effects that are difficult to maintain without additional supports, such as booster sessions.

Although BASIS was explicitly designed to isolate and influence individual-level processes, a wealth of implementation literature indicates that aspects of the inner organizational setting—such as organizational culture, climate, and leadership—are critical to implementation success [11, 14]. Although data collected in the current study demonstrated comparable implementation climate across the BASIS and control conditions, organizational issues may still have operated to decrease CBITS implementation in both groups. BASIS is not intended to be a stand-alone implementation strategy. Ultimately, we expect that combining individually oriented implementation strategies, such as BASIS, with organizational strategies (e.g., [13]) may have a synergistic effect on implementation and service outcomes.

It is also important to note that the majority of previous literature documenting significant effects of the TPB on clinician’s behavior has been conducted in relation to physical healthcare as opposed to mental health service delivery. In these studies, the desired behavior changes (e.g., prescribing, referring patients, performing an examination, documentation) sometimes differ in complexity from the multifaceted psychosocial interventions that typify mental health EBPs [76]. It is possible that it is particularly challenging to apply the TPB to enact change in mental health clinicians’ use of EBP, perhaps due to the complexity of the interventions. CBITS is a complex intervention with components that may limit its usability and ease of implementation. For instance, the first step to adopt CBITS involves administering screening procedures to detect youth who have experienced trauma and are in need of a trauma-informed intervention. Thus, initiating the delivery of CBITS involves significant motivation and effort on the part of the clinician to conduct trauma screening which involves sensitive questions and is not typical of routine practice in schools [58]. Additionally, because it is group-based, CBITS requires the coordination of many different student and staff calendars. Parent sessions, when conducted, add additional complexity.

### Applications of BASIS

Although BASIS did not demonstrate a sustained impact on proximal mechanisms through a 4-month follow-up, there were detectable improvements immediately following delivery. This suggests that BASIS may be a helpful strategy for generating an initial spike of energy at the beginning of an implementation process, a critical period when the introduction of new knowledge and behavior expectations may result in a short-term decrease in skill level [77]. This may be particularly useful for low complexity interventions. Alternatively, BASIS may have enough of an impact on TPB mechanisms to affect behavior change within a setting that is particularly conducive to new implementation efforts (e.g., favorable implementation leadership, climate, and citizenship behavior [69, 71, 78, 79]). In such settings, BASIS may be combined with—or integrated into—“cornerstone” implementation strategies such as training and post-training consultation efforts [80] to enhance their effects.

### Potential BASIS revisions

The findings suggest that, while the BASIS strategy was effective in shifting its proximal targets in the short term, additional supports may be necessary to ensure that gains persist over time and fully translate into changes in implementation behaviors. Previous research has demonstrated that change commitment fluctuates across the year, with overall decreasing trends [81], suggesting that behavioral intentions may change in response to a range of multilevel influences. BASIS was initially conceptualized as a pragmatic and time-limited implementation strategy, delivered immediately before and following training in an EBP. Revisions to the BASIS strategy that capitalize on its effectiveness in changing clinician perceptions and engagement in the short term, but increase its sustained impact, may include either (1) more explicit post-training barrier anticipation and action/coping planning and/or (2) incorporation of booster sessions to maintain shifts in attitudes, social norms, perceived behavioral control, and behavioral intentions.

#### Action and coping planning

Although BASIS buffered against decreases in implementation intentions following the training, these results highlight a potential need to develop BASIS components that more explicitly translate those intentions into implementation behaviors. The Health Adaption Process Approach (HAPA [82, 83]) is a stage model (in contrast to the TPB, which is considered a continuum model) that outlines processes of behavior change with the aim of minimizing the intention-behavior gap. HAPA distinguishes between a motivational phase, during which individuals develop their intentions to act, and a volitional phase, where those intentions are supported and transformed into actions. Action and coping planning are essential components of the volitional phase. Through *action planning*, individuals (e.g., clinicians) outline a clear and detailed plan of when and how they would implement the intended practice (e.g., CBITS). *Coping planning* allows clinicians to identify potential barriers that they expect to face in implementing the practice, and determine strategies for overcoming those barriers. Action and coping planning have been associated with higher levels of behavioral change [84]. Incorporating action and coping planning into BASIS will likely enhance clinicians’ abilities to set and pursue goals related to adoption and eventual high-fidelity delivery of CBITS.

#### Boosters

In addition, incorporating booster sessions to maintain positive shifts in proximal mechanisms may allow BASIS to facilitate adoption and delivery of the EBP over time once providers return to the realities of their jobs post-training. Boosters for specific EBPs have been shown to enhance adoption in school settings [85–88] and facilitate strong impacts on service recipient outcomes [89, 90]. BASIS booster sessions may revisit core BASIS components (e.g., strategic education to enhance attitudes toward EBPs), as well as provide an explicit opportunity for clinicians to adjust their action and coping plans according to the barriers and facilitators they have experienced thus far in their implementation. A booster session may be most effective during an active volitional phase, once clinicians have demonstrated strong intentions and some actions toward implementation, but may need continued support to persist with the delivery of the EBP [91, 92].

### Limitations

This research yielded findings that speak to the utility of an individual implementation strategy based on the TPB. There were also limitations that should be addressed by future research. This study was focused on collecting preliminary outcome data and evaluating BASIS feasibility in a complex service delivery setting. By design, this study was unable to test hypotheses related to (1) sustainment of change in TPB mechanisms, (2) impact on additional implementation outcomes (e.g., EBP fidelity), (3) impact on youth mental health outcomes, and (4) mediation models evaluating mechanisms of change. Additionally, although data were collected, this study was unable to control for relevant organizational context factors (e.g., implementation climate) when testing pre-post changes in proximal mechanisms due to power limitations. A fully powered randomized controlled trial of BASIS—potentially incorporating some of the revisions proposed above—could address these additional research questions within an effectiveness-implementation Hybrid Type 2 or Hybrid Type 3 design [93]. Considering the small sample size of this study, the findings presented should be interpreted with caution, especially for the few measure subscales that exhibited poor to moderate reliability. An emerging body of research, however, has demonstrated that Cronbach’s alpha may underestimate the true value of reliability, especially in cases with small sample sizes and scales with few items [94]. Regardless, it will be important for future research with more participants and more complex research designs to re-examine the mechanisms proposed in the current study.

## Conclusions

Existing implementation strategy compilations [36] contain few strategies explicitly designed to impact the individual-level mechanisms identified by the TPB (i.e., attitudes, social norms, perceived behavioral control, intentions). Further, most existing implementation strategies occur during the active implementation phase, rather than the preparation/pre-implementation phase. BASIS is a novel, pragmatic implementation strategy designed to address TPB constructs prior to the initiation of active implementation activities, such as training and consultation. The current study suggests that BASIS was effective in shifting its theorized mechanisms of change, but additional work is needed to confirm these findings and to revise BASIS to enhance and sustain its effects so clinicians are more responsive to consultative supports. Continued development and testing of pragmatic strategies, such as BASIS, is critical to efficiently support large-scale EBP implementation in the pursuit of promoting better patient outcomes.

## Additional file

Additional file 1: Detailed Measures Table. (DOCX 18 kb)

## Abbreviations

AC: Attention control
BASIS: Beliefs and Attitudes for Successful Implementation in Schools
CBITS: Cognitive Behavioral Intervention for Trauma in Schools
EBP: Evidence-based practice
EBPAS: Evidence-Based Practice Attitudes Scale
HAPA: Health adaption process approach
SICBS: School Implementation Citizenship Behavior Scale
TPB: Theory of planned behavior
