# Supplementary material for: The influence of a blended, theoretically-informed pre-implementation strategy on school-based clinician implementation of an evidence-based trauma intervention
Source: Implement Sci. 2019 May 30;14:54. doi: 10.1186/s13012-019-0905-3 (PMC6543642; doi:10.1186/s13012-019-0905-3)
Supplement: Supplementary file 1 — Detailed Measures Table. (DOCX 18 kb) [file 13012_2019_905_MOESM1_ESM.docx]

Supplemental File 1. Detailed Measures Table.

| Construct | Instrument and Description |
| --- | --- |
| Attitudes | The school-adapted version of the Evidence-Based Practice Attitudes Scale (EBPAS) [64] is a 26-item adaptation of the original EBPAS [65]. Items are rated on a five-point scale (0 = “Not at All” to 4 = “To a Very Great Extent”). Internal reliabilities are adequate for the total score (alpha = .94) and the subscale scores: (1) likelihood of adopting if the EBP were a requirement (3 items, α = .81), (2) likelihood of adopting if the EBP were appealing to you (4 items, α = .87), (3) openness to new practices (4 items, α = .82), (4) perceived divergence between current practices and EBPs (4 items, α = .39), (5) fit of EBPs with current practices (7 items, α = .84), and (6) burden of EBPs (4 items, α = .66). Attitudes were measured at baseline, post, and follow-up time points. |
| Perceived Behavioral Control / Self-Efficacy | A modified version of the Teacher Self-Efficacy Scale [68] has 10 items assessing perceived behavioral control in implementing EBP. Item wording was modified to be consistent with self-efficacy as it relates to the implementation of EBPs. This scale assesses teachers’ confidence and self-efficacy regarding teaching practices. Items are rated on a four-point scale (1 = “Not true at all”, 2 = “Hardly true”, 3 = “Moderately true”, 4 = “Very true”). The measure has demonstrated sufficient internal consistency (alpha = .82), test-retest reliability (r = .65 for over two year), and criterion validity; in our study, we found very high internal consistency at baseline (alpha = .94). Perceived behavioral control was measured at baseline, post, and follow-up time points. |
| Perceived Social Norms | The modified Subjective Norms measure, used in previous studies and based on guidelines for developing reliable and valid measures of Theory of Planned Behavior (TPB) constructs, [66,67] captures two types of EBP implementation-related subjective norms: injunctive and descriptive. Injunctive norms are the perception of what ought to be or what the social group would approve of (e.g., “Others who I respect expect me to adopt and implement evidence-based practices that promote students’ social, emotional, and behavioral functioning”). Injunctive norm items (4 items, α = .57) are rated on a seven point scale (-3 = “I should”, 0 = “Neutral”, 3 = “I should not”). Descriptive norms describe perceptions of how the social group actually does things (e.g., “Practitioners like me find the time to implement evidence-based practices”). Descriptive norm items (4 items, α = .71) were rated on a seven-point scale (-3 = “Strongly agree”, 0 = “Neutral”, 3 = “Strongly disagree”). Perceived social norms were measured at baseline, post, and follow-up time points. To aid interpretation, we have reversed the original scoring of the measure so that more positive scores represent higher levels of subjective norms. |
| Implementation Citizenship | The School Implementation Citizenship Behavior Scale (S-ICBS) [69], modified from the original Implementation Citizenship Behavior Scale [70] measures clinicians’ perceptions regarding how school staff engage with EBPs within their specific school context. The S-ICBS includes 15 items (total α = .94) across 4 subscales: (1) Helping others (3 items, e.g., “School staff assist others to make sure they implement evidence-based practices properly”, α = .86), (2) Keeping informed (3 items, e.g., “School staff keep informed of changes in evidence-based practice policies and procedures”, α = .83), (3) Taking Initiative (4 items, e.g., “School staff willingly take on additional responsibilities related to the implementation of evidence-based practice”, α = .92), and (4) Advocacy (e.g., 5 items, e.g., “School staff here are proponents of evidence-based practice.”, α = .90). All items were reported on a 5-point scale (0 = *not at all*, 4 = *frequently, if not always*). Implementation citizenship was measured at baseline, post, and follow-up time points. |
| Behavioral Intentions | The Modified Intentions to Use Scale [71] assesses school mental health providers’ intentions to implement EBP. This scale was developed based on established guidelines for developing behavioral intention measures using the TPB [66,67]. The scale includes 9 items (α = .90; e.g., “I plan to use evidence-based practices with students”) rated on a 5 point scale (0 = “Not at all”, 4 = “To a very great extent”). This scale was administered pre and post BASIS and AC conditions to examine changes in school mental health providers’ intentions to implement CBITS. |
| BASIS Fidelity | A fidelity tool was developed by the research team to capture dimensions of adherence to BASIS components. Across both pre- and post-training BASIS sessions, 33 components (e.g., ‘engage participants in generating solutions to resolve the time barrier’) were rated as present or not present. These items were rated on a Likert scale (0 = *not at all* to 4 = *very great extent*). Both conditions (AC and BASIS) were videotaped and independently coded for fidelity. Two coders independently rated the BASIS condition; one rater coded 97% and the second rater coded 94% of 33 BASIS components as having been delivered. The raters failed to jointly classify any component as not delivered. In situations such as this, Cohen’s kappa statistic is an inappropriate measure of interrater reliability [73]. Therefore, we report that raters were in agreement on 91% of the components delivered in the BASIS condition, and that a minimum of 91% of components were delivered during BASIS. Only one rater coded the AC condition; it covered 21% of BASIS components. |
| Consultation Engagement | Each participant was provided the opportunity to attend up to 13 consultation phone calls with a CBITS consultant. At the end of each call the consultant filled out an electronic survey pertaining to participant engagement in the calls. The consultant recorded who attended the consultation call and whether or not homework had been assigned on the previous call and any participants who had completed that assignment. Using a five-point Likert scale (1= Not at all to 5=Fully engaged), the consultant was then asked to rate each participant’s engagement in that specific consultation call. Ongoing participation was operationalized by a number of indicators, including (1) attending at least one post-training consultation session, (2) number (%) of consultation sessions attended, (3) number of days post-training until consultation dropout, defined by verbal/written withdrawal from consultation, or 3 consecutive missed consultation sessions. |
| Adoption | Adoption is operationalized as the initiation of a CBITS group at any point during study participation, the number of days until the first CBITS session. Non-adoption is operationalized as the number of days until implementation dropout (i.e., when the participant declared that they were not going to implement CBITS). Previous research indicates that the vast majority of CBITS groups, once initiated, are completed. [43] |
| Implementation Climate | The school-adapted Implementation Climate Scale (ICA) [69,72] provides a method to rate schools’ contextual preparedness for and strategic support of the implementation of specific innovations such as an EBP. It measures clinician perceptions of the extent to which EBP implementation is expected, supported, and rewarded in their setting. The measure features 21 items with 7 subscales. Focus on EBP, Educational Support for EBP, Recognition for EBP, Rewards for EBP, Use of Data to support EBP, Use of Existing Supports/Infrastructure for EBP Implementation, and Degree of EBP Integration (into routines). Subscale internal consistency ranges from .85 to .97 [69]. The ICA correlates moderately with, but is distinct from, a conceptually similar strategic climate measure. While implementation climate as a construct exists at the organization level, is generally measured by aggregating responses from a wide range of participants within the organization. Furthermore, because the construct of climate reflects individuals’ reactions to the context of a particular organization, climate perceptions may also be measured at the individual level [98]. Due to the difficulty of collecting data from all staff at each school, including those not participating in the study, we treat this as individual-level data for the current study. |
